# The crystal structure of mycothiol disulfide reductase (Mtr) provides mechanistic insight into the specific low-molecular-weight thiol reductase activity of Actinobacteria

**DOI:** 10.1107/S205979832400113X

**Published:** 2024-02-19

**Authors:** Javier Gutiérrez-Fernández, Hans-Petter Hersleth, Marta Hammerstad

**Affiliations:** a Section for Biochemistry and Molecular Biology, Department of Biosciences, University of Oslo, PO Box 1066, Blindern, 0316 Oslo, Norway; b Centre for Molecular Medicine Norway, Nordic EMBL Partnership, University of Oslo, 0318 Oslo, Norway; University of Sâo Paulo, Brazil

**Keywords:** low-molecular-weight thiols, mycothiol disulfide reductase, oxidoreductases, redox homeostasis, flavoenzymes, X-ray crystallography, docking, protein structure, Actinobacteria

## Abstract

Low-molecular-weight (LMW) thiols are involved in many processes in all organisms, playing a protective role against reactive species, heavy metals, toxins and antibiotics. Actinobacteria, such as *Mycobacterium tuberculosis*, use the LMW thiol mycothiol (MSH) to buffer the intracellular redox environment. The NADPH-dependent FAD-containing oxidoreductase mycothiol disulfide reductase (Mtr) is known to reduce oxidized mycothiol disulfide (MSSM) to MSH, which is crucial to maintain the cellular redox balance. In this work, the first crystal structures of Mtr are presented, expanding the structural knowledge and understanding of LMW thiol reductases. The structural analyses and docking calculations provide insight into the nature of Mtrs, with regard to the binding and reduction of the MSSM substrate, in the context of related oxidoreductases. The putative binding site for MSSM suggests a similar binding to that described for the homologous glutathione reductase and its respective substrate glutathione disulfide, but with distinct structural differences shaped to fit the bulkier MSSM substrate, assigning Mtrs as uniquely functioning reductases. As MSH has been acknowledged as an attractive antitubercular target, the structural findings presented in this work may contribute towards future antituberculosis drug development.

## Abstract 2

The crystal structure of mycothiol disulfide reductase (Mtr) was determined for the first time. The structure shows a highly conserved and enlarged substrate-binding pocket, providing insight into the substrate-binding mode and specificity of Mtrs.

## 1. Introduction

Eukaryotes, most Gram-negative bacteria and some Gram-positive bacteria use the well studied glutathione (GSH) as their major low-molecular-weight (LMW) thiol (Fahey *et al.*, 1978 ▸; Loi *et al.*, 2015 ▸). LMW thiols are a group of reactive sulfhydryl-containing compounds that play critical roles as intracellular redox buffers that maintain cytosolic proteins in their reduced states and in protection against, for example, oxygen and antibiotic toxicity. Most Gram-positive bacteria do not produce GSH and rely on alternative thiol-redox buffers. Firmicutes (low-G+C Gram-positive bacteria), including *Staphylococcus aureus* and many bacilli, utilize bacillithiol (BSH) to protect the cells against a variety of reactive species (Newton *et al.*, 2009 ▸; Sharma *et al.*, 2011 ▸; Gaballa *et al.*, 2010 ▸), whereas in most Actinobacteria (high-G+C Gram-positive bacteria), such as mycobacteria, *Streptomycetes* and corynebacteria, mycothiol (MSH) serves as the predominant LMW thiol (Newton *et al.*, 1996 ▸; Reyes *et al.*, 2018 ▸; Sakuda *et al.*, 1994 ▸; Spies & Steenkamp, 1994 ▸). Many Actinobacteria are important in human pathogenesis (for example *Mycobacterium tuberculosis*, the causative agent of tuberculosis) and the detoxification of contaminants (for example *Rhodococcus*) (Nazari *et al.*, 2022 ▸). MSH, formed by the conjugation of *N*-acetylcysteine with 1-d-*myo*-inosityl-2-amido-2-deoxy-α-d-glucopyranoside (Newton *et al.*, 1995 ▸; Jothivasan & Hamilton, 2008 ▸) and present at millimolar concentrations in the cell (Newton *et al.*, 1996 ▸), plays analogous roles to those of GSH in maintaining cellular redox homeostasis. One such role includes redox regulation of proteins and protection of protein thiols through the formation (termed *S*-mycothiolation) of mixed disulfides between MSH and protein thiols that are formed under conditions of oxidative stress (Chi *et al.*, 2014 ▸; Reyes *et al.*, 2018 ▸). Additionally, MSH participates in a variety of metabolic processes, such as protection against heavy metals, alkylating agents, reactive oxygen species (ROS) and reactive nitrogen species (RNS), as well as detoxification of antibiotics and electrophiles (Loi *et al.*, 2015 ▸). Under oxidative stress conditions, MSH is oxidized to the disulfide (MSSM) state. For instance, *S*-mycothiolation is redox-controlled by the gluta­redoxin homolog mycoredoxin-1 (Mrx1), which regenerates enzyme activity through the reduction of *S*-mycothiolated proteins, resulting in an Mrx1-SSM intermediate that is reduced by MSH, and ultimately leading to the formation of MSSM (Van Laer *et al.*, 2012 ▸). Similarly, GSH is oxidized to glutathione disulfide (GSSG) and BSH is oxidized to bacilli­thiol disulfide (BSSB). In order to maintain intracellular redox homeostasis, the disulfide forms are reduced back to the reduced states by the respective flavoprotein disulfide reductases (FDRs): GSSG by glutathione reductase (GR) and BSSB by bacillithiol disulfide reductase (Bdr) (Fahey *et al.*, 1978 ▸). The lack of GR activity in mycobacterial cell lysates suggested the occurrence of a distinct reductase in these bacteria that is functionally analogous to GR but that exhibits no activity for GSSG and has been shown to have an absolute requirement for the glucosamine moiety of the MSSM substrate for activity (Patel & Blanchard, 1998 ▸, 1999 ▸). To restore the MSH/MSSM redox balance, Actinobacteria encode the flavoenzyme mycothiol disulfide reductase (Mtr; also called mycothione reductase), which catalyzes the reduction of MSSM to MSH, as demonstrated for *Mycobacterium smegmatis* Mtr (Patel & Blanchard, 1998 ▸) and *M. tuberculosis* Mtr (Patel & Blanchard, 1999 ▸; Kumar *et al.*, 2017 ▸), and was originally characterized using a truncated substrate. Mtr belongs to a group of NAD(P)H-dependent homodimeric oxidoreductases with a tightly bound flavin adenine dinucleotide (FAD) cofactor per subunit. The overall structure composed of two dinucleotide-binding domains (the ‘two dinucleotide-binding domains’ flavoproteins; tDBDFs), which are responsible for binding FAD and NAD(P)H, respectively, is seen in many of these oxidoreductases, and is commonly described for the low-molecular-weight (low *M*_r_) thioredoxin reductases (TrxRs), as typified by the *Escherichia coli* enzyme (Argyrou & Blanchard, 2004 ▸; Williams, 1995 ▸). In addition, each subunit contains a catalytic redox-active disulfide/Cys pair that is responsible for the reduction of their respective and structurally unique disulfide-bonded substrates. Although some flavin-dependent oxidoreductases are solely composed of the low *M*_r_ TrxR architecture, other oxido­reductases contain the low *M*_r_ TrxR architecture with additional domains making up larger structures, as in the case of, for example, high *M*_r_ TrxR and GR (Hammerstad & Hersleth, 2021 ▸; Kuriyan, Krishna *et al.*, 1991 ▸). Oxidoreductases of this group all share conserved amino-acid sequence motifs for the binding of NAD(P)H and FAD, and a characteristic His–Glu ion pair that is involved in proton transfer, and most share a similar catalytic mechanism (Patel & Blanchard, 1999 ▸, 2001 ▸; Fagan & Palfey, 2010 ▸). The initial characterization of Mtr suggested a mechanism similar to that of the prototypical GR, exhibiting a bi-bi ping-pong kinetic mechanism (Patel & Blanchard, 1999 ▸; Massey & Williams, 1965 ▸). In the reductive half-reaction, a two-electron-reduced enzyme is generated through the reduction of the redox-active Cys pair by NADPH, via FAD. Subsequent electron transfer of the reducing equivalents in turn reduces the disulfide substrate through a dithiol–disulfide interchange step in the oxidative half-reaction. From a structural perspective, in GR electrons flow from NAD(P)H, bound on the *re* face of the FAD isoalloxazine ring, to the Cys pair on the *si* face of FAD, where the now reduced dithiol reacts with GSSG. Although similar in sequence to GR, the rate of the oxidative half-reaction was shown to be slightly faster than the reductive half-reaction in Mtr, unlike as is seen in most FDRs, where the oxidative half-reaction is commonly rate-limiting (Patel & Blanchard, 2001 ▸). More knowledge on the catalytic mechanism could potentially be attained from a crystal structure. Evidence that MSSM is recycled by the FAD-containing NAD(P)H-dependent oxidoreductase Mtr has provided insight into MSH-dependent mechanisms and the Mrx1/MSH/Mtr pathway in Actinobacteria; however, detailed structural data on Mtrs have been lacking to date. A low-resolution small-angle X-ray scattering (SAXS) solution structure of Mtr confirmed the dimeric state of the enzyme, however, indicating the presence of an asymmetric dimer (Kumar *et al.*, 2017 ▸). A crystal structure of Mtr would provide a missing link in the LMW thiol field and an important addition to the previously characterized crystal structures of LMW thiol-specific reductases such as GR (Karplus & Schulz, 1989 ▸), Bdr (Hammerstad *et al.*, 2020 ▸) and coenzyme A disulfide reductase (CoADR; Mallett *et al.*, 2006 ▸). How structurally similar are Mtrs to related oxidoreductases? Can a crystal structure provide insight into the mechanism of MSSM reduction by Mtr, as compared with GSSG reduction by GR? Furthermore, MSH is the major LMW thiol involved in the maintenance of redox balance crucial for the survival of the human pathogen *M. tuberculosis*, and has been shown to contribute to its pathogenicity, infection and antibiotic-resistance mechanisms (Rawat *et al.*, 2002 ▸; Trivedi *et al.*, 2012 ▸; Nambi *et al.*, 2015 ▸; Sareen *et al.*, 2003 ▸; Sassetti & Rubin, 2003 ▸). MSH has already been recognized as an attractive antitubercular target (Nilewar & Kathiravan, 2014 ▸); however, with an increasing number of drug-resistant *M. tuberculosis* strains, new structural and mechanistic insight into enzymes involved in MSH redox biology, including Mtr, could provide valuable information for future antituberculosis drug development. Moreover, the ability of Mtr from *Rhodococcus erythropolis* to reduce tellurite ( 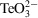 ) to elemental tellurium (Butz *et al.*, 2021 ▸), as also reported for other reductases (Moore & Kaplan, 1992 ▸; Maltman *et al.*, 2017 ▸; Arenas-Salinas *et al.*, 2016 ▸), makes Mtr an interesting candidate for bioremediation research as well as for nanotechnology involving tellurium-based nano­structures.

In this work, we present the first reported crystal structures of Mtr from two homologous Actinobacteria: *R. erythropolis* and the close *M. tuberculosis* relative *M. smegmatis*. The overall structural architecture of homodimeric Mtr highly resembles that of the well characterized flavin-dependent oxido­reductase GR. Using docking calculations and inspection of the Mtr structures, as well as comparison with GR, we propose a putative binding site for the MSSM substrate. Our findings demonstrate that MSSM can bind placing its disulfide bond in the proximity of the FAD cofactor and redox-active Cys pair in Mtr, allowing reduction of the substrate. Although Mtrs and GRs share a similar catalytic mechanism and substrate-binding site, we have identified structural differences that are likely to account for the substrate specificity for MSSM in Mtrs. The highly conserved MSSM binding site in Mtr is considerably larger than in GR, in agreement with its bulkier natural substrate, making Mtrs distinct and unique in terms of function. This study provides an important missing link in the field of redox biology and LMW thiols, as well as NAD(P)H-dependent FAD-containing oxidoreductases, providing new insight into the biological function of Mtrs.

## 2. Materials and methods

### 2.1. Expression and purification of Mtr*_Re_* and Mtr*_Ms_*

The genes for *R. erythropolis* PR4 Mtr (Mtr*_Re_*; locus tag RER_26020) or for *M. smegmatis* MC^2^ 155 Mtr (Mtr*_Ms_*; locus tag LJ00_12995) were cloned into a pET-22b(+) plasmid (constructed using NdeI and XhoI sites; GenScript) and transformed into competent *E. coli* One Shot BL21 (DE3) cells (Invitrogen, Thermo Fisher Scientific). The cells were grown in LB medium containing 100 µg ml^−1^ ampicillin. Protein expression was induced by adding isopropyl β-d-1-thiogalactopyranoside (IPTG) to a final concentration of 0.5 m*M* on reaching an OD_600 nm_ of 0.4–0.6, and the cultures were incubated at 15°C for 24 h with shaking before the cells were harvested and stored at −80°C.

The cells were thawed, dissolved in 50 m*M* Tris–HCl pH 7.5, 1 m*M* DTT, 5 µg ml^−1^ DNase with a cOmplete Protease Inhibitor Cocktail tablet (Roche) at a 1:10 cell wet weight:buffer ratio and lysed by sonication. Mtr*_Re_* and Mtr*_Ms_* were precipitated with 0.6 and 0.4 g ml^−1^ ammonium sulfate, respectively, dissolved in 50 m*M* Tris–HCl pH 7.5, 1 m*M* DTT and desalted by dialysis (SnakeSkin dialysis tubing, 10K molecular-weight cutoff, Thermo Fisher Scientific). The desalted proteins were filtered through a 0.45 µm filter (Merck Millipore), applied onto a HiTrap Q FF anion-exchange column (Cytiva) and eluted with a linear gradient to 50 m*M* Tris–HCl pH 7.5, 1 m*M* DTT, 1 *M* NaCl. Finally, the proteins were purified on a Superdex 200 Increase 10/300 GL column (Cytiva) in 50 m*M* HEPES pH 7.5, 150 m*M* NaCl. The eluted Mtr*_Re_* and Mtr*_Ms_* both showed approximate molecular weights of 100 kDa, corresponding to the dimeric form of Mtr, as validated by calibration of the size-exclusion column (Gel Filtration LMW Calibration Kit, Cytiva). Protein fractions were pooled and concentrated to 30 mg ml^−1^ using Amicon Ultra-15 filter units (50 kDa molecular-weight cutoff, Merck Millipore), flash-frozen in liquid nitrogen and stored at −80°C. All chromatographic steps were performed on an ÄKTApurifier FPLC system (GE Healthcare) and all expression and purification steps were monitored by sodium dodecyl sulfate–polyacrylamide gel electrophoresis (SDS–PAGE). UV–visible (UV–Vis) spectra were recorded on an Agilent Cary 60 spectrophotometer and the protein concentrations were estimated using an extinction coefficient at λ_max_ of ɛ_465 nm_ = 11.5 m*M* ^−1^ cm^−1^.

### 2.2. Protein crystallization and structure determination

Initial crystallization screening of both Mtr proteins was performed using the sitting-drop vapor-diffusion crystallization method with a Mosquito robot (SPT Labtech). Initial hits giving rod-shaped crystals of Mtr*_Re_* (30 mg ml^−1^) of approximately 100 × 50 × 50 µm in size were obtained with the MIDASplus screen from Molecular Dimensions and were further optimized in 45%(*w*/*v*) polypropylene glycol 400, 4%(*v*/*v*) ethanol. Rod-shaped crystals of Mtr*_Ms_* (12.5 mg ml^−1^) of approximately 200 × 30 × 30 µm in size were obtained with Index from Hampton Research [0.02 *M* magnesium chloride hexahydrate, 0.1 *M* HEPES pH 7.5, 22%(*w*/*v*) poly(acrylic acid sodium salt) 5100]. The crystals were grown at 20°C, cryoprotected in 50%(*w*/*v*) polypropylene glycol 400 [and 4%(*v*/*v*) ethanol for Mtr*_Re_*] and flash-cooled in liquid nitrogen.

X-ray diffraction data were collected at 100 K on beamlines ID23-2 and ID30B for Mtr*_Re_* and Mtr*_Ms_*, respectively, at the European Synchrotron Radiation Facility (ESRF), Grenoble, France. Diffraction images were processed with *XDS* (Kabsch, 2010 ▸) and *AIMLESS* (Evans, 2011 ▸; Agirre *et al.*, 2023 ▸) to resolutions of 2.9 Å for Mtr*_Re_* and 4.7 Å for Mtr*_Ms_*. The structures contained two molecules per asymmetric unit. The structure of Mtr*_Re_* was solved by molecular replacement using *Phaser* (McCoy *et al.*, 2007 ▸) with an *AlphaFold* (Jumper *et al.*, 2021 ▸) *ab initio* model of *R. erythropolis* DN1 Mtr as a template (pLDDT > 90), and the solved structure was further used as a template to solve the Mtr*_Ms_* structure, in which the chains were rebuilt with *CHAINSAW* (Stein, 2008 ▸). Several rounds of rebuilding and various refinement strategies were performed using *Coot* (Emsley *et al.*, 2010 ▸) and *Phenix* (Liebschner *et al.*, 2019 ▸). The Mtr*_Re_* data were twinned, and the twin law *h*, −*h* − *k*, −*l* with a twin fraction of 0.47 was used in all refinements in *Phenix*. The best Mtr*_Re_* structure was obtained by refining *XYZ* in both reciprocal and real space, translation–libration–screw (TLS) rotation factors and individual *B* factors, and applying noncrystallographic symmetry (NCS) restraints. A similar refinement was performed for Mtr*_Ms_* but using isotropic *B* factors and no TLS rotation factors. For the Mtr*_Ms_* structure, a round of refinement with *PDB-REDO* was performed (Joosten *et al.*, 2014 ▸). The presence of disulfide bonds between the redox-active Cys pairs was confirmed in both chains in Mtr*_Re_* by omit maps, although some minor negative electron density was present indicating slightly incomplete occupancy of the disulfide bridges. *PDBePISA* was used to analyze the buried surface area of the Mtr dimer. The absorbed X-ray dose was calculated using *RADDOSE-*3*D* (Zeldin *et al.*, 2013 ▸). All structural figures were prepared with *PyMOL* version 2.5 (Schrödinger).

### 2.3. Docking analysis and protein–ligand interactions

Docking studies between Mtr*_Re_* and MSH or MSSM were performed with *CB-Dock*2 (*Cavity-detection guided Blind Docking*; Liu *et al.*, 2022 ▸). Structure-based blind-docking calculations were performed using the dimeric structure of Mtr*_Re_* and the experimental MSH structure from *M. tuberculosis* mycothiol *S*-transferase (PDB entry 8f5v; Jayasinghe *et al.*, 2023 ▸) or an MSSM model, with human GR with GSSG bound (PDB entry 1gra; Karplus & Schulz, 1989 ▸) as a reference model for template-based blind docking. To generate schematic diagrams of protein–ligand interactions, *LigPlot*+ (Wallace *et al.*, 1995 ▸; Laskowski & Swindells, 2011 ▸) was used, applying a 4 Å cutoff radius, to show hydrogen bonds and hydrophobic contacts, which are represented by dashed lines and by arcs with spokes radiating towards the ligand atoms that they contact, respectively.

### 2.4. Bioinformatics analysis: sequence-similarity networks, multiple sequence alignment and phylogenetic analysis

Sequence-similarity networks (SSNs) were generated with the EFI Enzyme Similarity Tool EFI-EST (https://efi.igb.illinois.edu/efi-est/; Oberg *et al.*, 2023 ▸), using *M. tuberculosis* Mtr as a search sequence, in order to analyze the relation to other similar oxidoreductases. The search included bacterial, archaeal and eukaryotic taxonomic groups using the UniRef50 and UniRef90 sequence databases, retrieving a total of 16 589 homologous sequences. Sequences were grouped with an alignment score of 120 and nodes representing sequences sharing >60% identity, dividing the main homologous oxidoreductases into separate clusters. Figures illustrating SSN analyses were created in *Cytoscape* (version 3.9) with the organic layout (Shannon *et al.*, 2003 ▸). The ten largest clusters of sequences were selected and assembled into seven groups with respect to their annotated function. Multiple sequence alignments were performed in *JalView* (Waterhouse *et al.*, 2009 ▸) with *Clustal Omega* (Sievers *et al.*, 2011 ▸) and phylo­genetic tree analysis with average distances using the BLOSUM62 matrix on two or three selected sequences from each of the ten clusters, including the sequences for the top *DALI* hits.

### 2.5. Structure comparison: structural alignment search with *DALI*

A search for similar structures to Mtr in the Protein Data Bank (PDB) was performed with the *DALI* (*Distance-matrix ALIgnment*) protein structure-comparison server (Holm, 2022 ▸) using the Mtr*_Re_* structure as a search template. For the top selected hits, a multiple structure sequence alignment was generated with *DALI* including secondary-structure assignments by *DSSP* (Kabsch & Sander, 1983 ▸; Touw *et al.*, 2015 ▸) through *DALI* and was presented with *JalView*.

### 2.6. Analysis of conserved residues

The degree of conservation of residues in the Mtr*_Re_* and GR (PDB entry 1gra; Karplus & Schulz, 1989 ▸) structures was evaluated with *ConSurf* (Ashkenazy *et al.*, 2010 ▸, 2016 ▸; Celniker *et al.*, 2013 ▸). The analysis was based on identification of homologous sequences from the UniRef90 database using the *HMMER* algorithm (Finn *et al.*, 2015 ▸) and multiple sequence alignment with *MAFFT* (Katoh & Standley, 2013 ▸). Of the homologous sequences passing the standard threshold, *ConSurf* selected a sample of 150 representative sequences. The sequences were inspected in *JalView* and all 150 sequences from the GR search were annotated as GRs, while only 97 of the 150 sequences selected from the Mtr search were annotated as Mtrs. Therefore, *ConSurf* was re-run using the 97 Mtr sequences to obtain a conservation degree based only on annotated Mtrs. A nine-bin colored scale was used to show the conservation of each residue, from most variable (turquoise) to most conserved (maroon), when generating three-dimensional figures with *PyMOL* or coloring the residues in *LigPlot*+.

## 3. Results and discussion

### 3.1. Overall structures of Mtr*_Re_* and Mtr*_Ms_*

**Figure 1:**
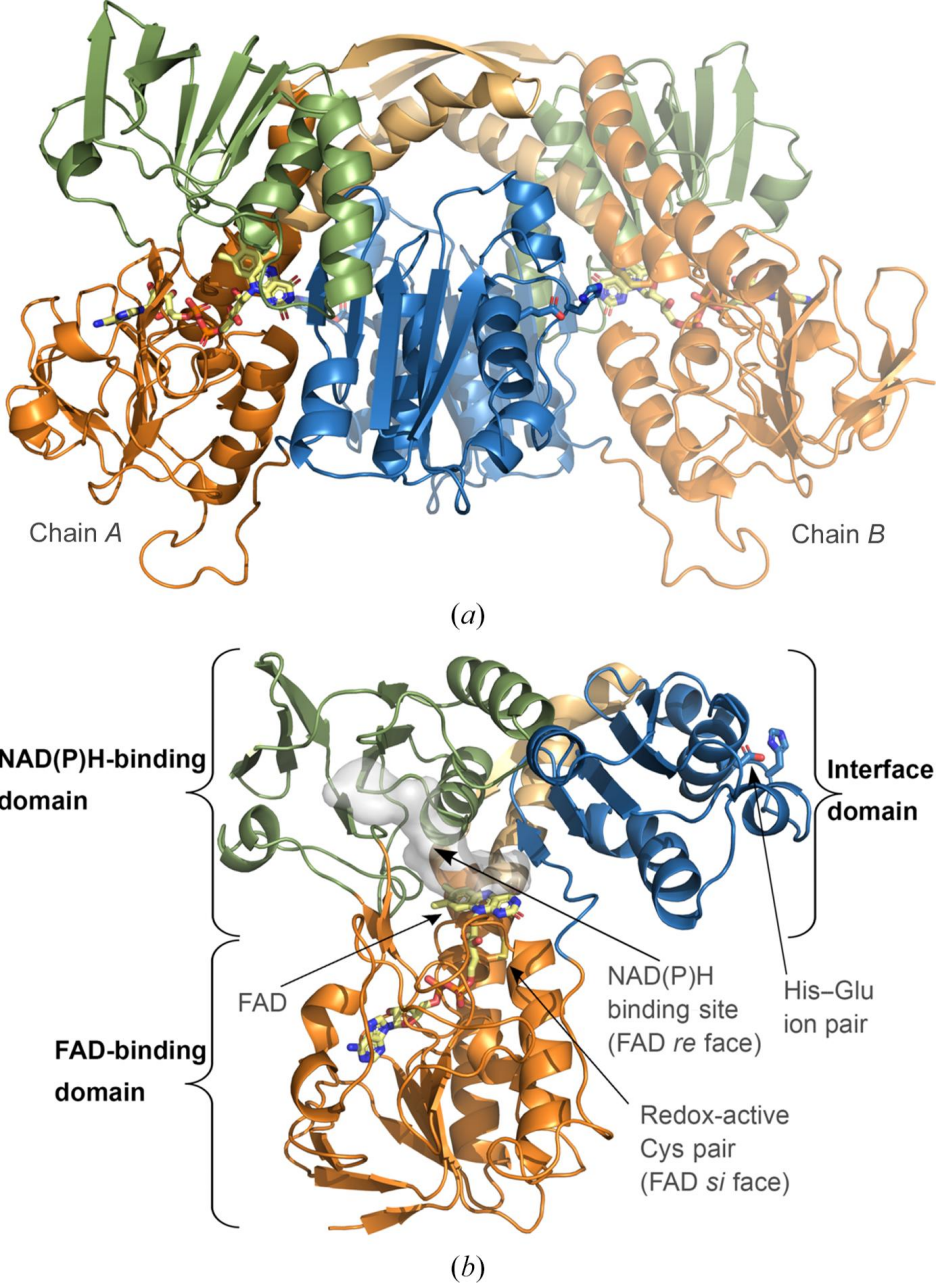
Crystal structure of Mtr*_Re_*, showing the biological dimer in (*a*) and a single monomer (chain *A*) in (*b*), displaying the FAD-binding domain in orange, the NAD(P)H-binding domain in green and the interface domain in blue. In (*b*), additional structural features are indicated. The FAD cofactor and catalytic residues are represented as sticks and colored by atom type.

Two structures of Mtrs from homologous Actinobacteria are presented in this work; Mtr*_Ms_* from the *M. tuberculosis* model organism *M. smegmatis* and Mtr*_Re_* from *R. erythropolis*, a biocatalyst used for the bioremediation of toxic compounds. The crystal structures of Mtr*_Re_* and Mtr*_Ms_* confirm that Mtr is a homodimer, as also supported by molecular-weight estimations during protein purification. Each monomer, composed of 458 and 461 residues in Mtr*_Re_* and Mtr*_Ms_*, respectively, belongs to the pyridine nucleotide-disulfide oxidoreductase (PNDO) superfamily (Lu *et al.*, 2020 ▸) and consists of three domains, namely a NAD(P)H-binding domain, a FAD-binding domain and a dimerization/interface domain (Fig. 1 ▸). In FAD-containing NAD(P)H-dependent oxidoreductases, two globular dinucleotide-binding three-layer ββα-sandwich Rossmann-like fold domains are fused into a single chain responsible for the binding of FAD and NAD(P)H (Ojha *et al.*, 2007 ▸) through conserved amino-acid sequence motifs (Susanti *et al.*, 2017 ▸; Dym & Eisenberg, 2001 ▸; Hammerstad & Hersleth, 2021 ▸), as also seen in the Mtr structures. Insight into the dimeric state of Mtr from *M. tuberculosis* has previously been given by Grüber and coworkers, demonstrated by a low-resolution structure derived from SAXS data, as well as dynamic light-scattering (DLS) studies (Kumar *et al.*, 2017 ▸). The latter study, however, proposed an extended conformation of the NAD(P)H-binding domain of one of the monomers, resulting in an asymmetric dimer assembly which indicates domain flexibility in solution. This feature is not seen in crystal structures of other homologous oxidoreductases, or in the Mtr*_Re_* or Mtr*_Ms_* crystal structures, which are both composed of a symmetrical dimer assembly.

The crystal contacts between the monomers were analyzed with *PISA* using the Mtr*_Re_* structure, showing a total buried surface area of 3405 Å^2^, which is in strong agreement with the total interface area calculated for the dimeric GR (Karplus & Schulz, 1987 ▸). Moreover, the total solvent-accessible surface area of Mtr is 34 240 Å^2^, the complex-formation significance score (CSS) is 0.668 and the solvation free-energy gain upon the formation of the interface (Δ^i^ *G*) is −38.0 kcal mol^−1^. Its formation entails a solvation free energy (Δ*G* ^int^) of −51.6 kcal mol^−1^, while the free energy of dissociation (Δ*G* ^diss^) is 42.2 kcal mol^−1^. Therefore, the dimeric Mtr crystal structure shown in this work strongly corresponds to the *in vivo* biological assembly.

**Figure 2:**
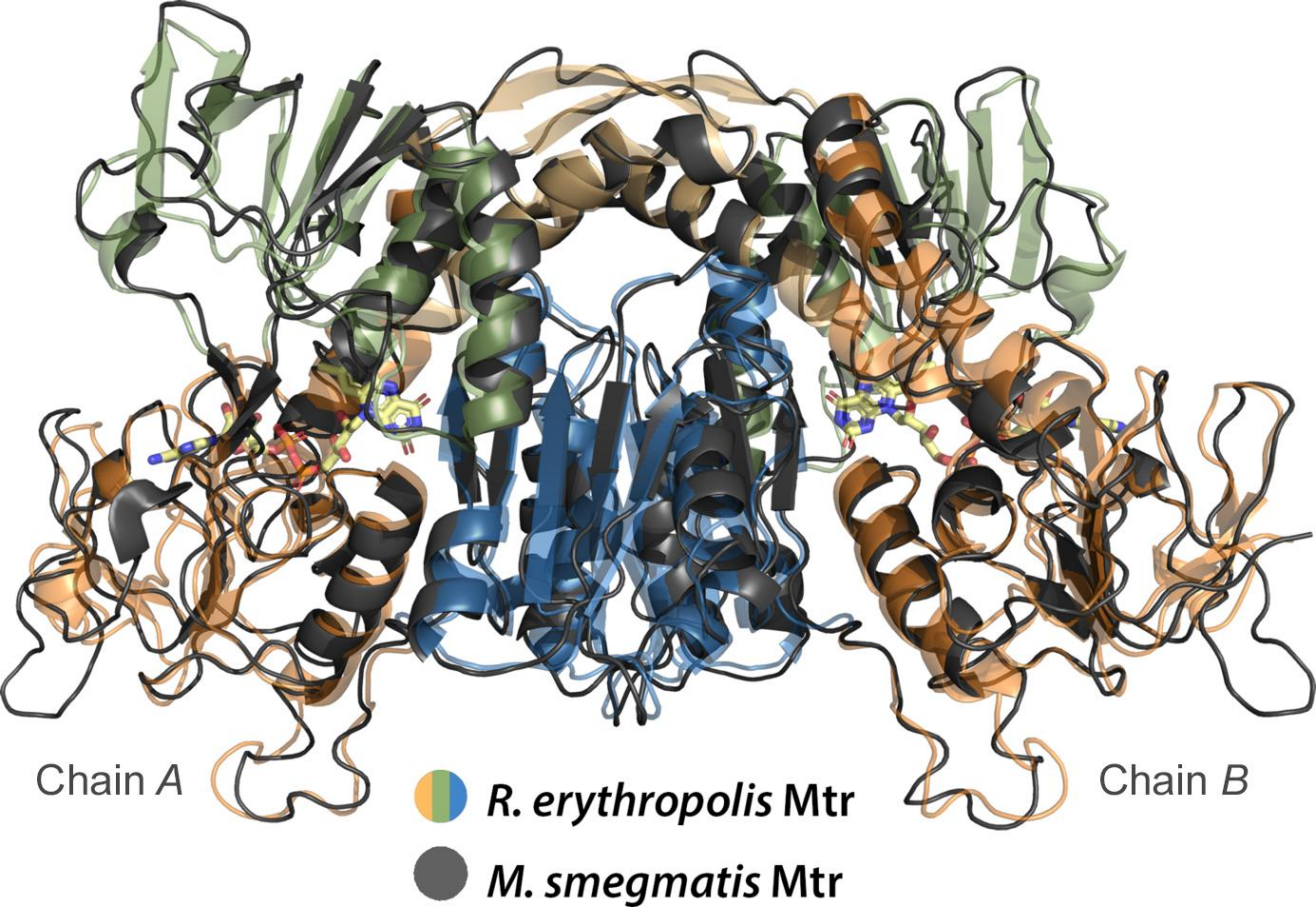
Structural alignment of Mtr*_Re_* (multicolored) and Mtr*_Ms_* (gray), showing the similar overall fold of the biological dimers of the two crystal structures. The FAD cofactor corresponds to Mtr*_Re_* and is shown as sticks and colored by atom type.

Little deviation is observed between Mtr*_Re_* (2.9 Å resolution) and Mtr*_Ms_* (4.7 Å resolution), which show highly similar overall folds with an r.m.s.d. of 1.0 Å (Table 1 ▸ and Fig. 2 ▸). Despite the low resolution obtained for the latter structure, it is clear that it adopts the structurally conserved topology that is seen for members of the PNDO superfamily and that major features are invariant between the two structures in this study. In addition, a strongly comparable *A*_465 nm_/*A*_280 nm_ ratio for both proteins, as well as the yellow crystals obtained of Mtr*_Re_* and Mtr*_Ms_*, support the presence of FAD in both proteins, although FAD could not be built into the Mtr*_Ms_* structure due to its low resolution and lack of significant density. Together, these results demonstrate that the detailed structural features observed and described for the structure of Mtr*_Re_* are representative of both Mtr*_Re_* and Mtr*_Ms_*, as well as other Mtrs across species (Fig. 2 ▸). In Mtr*_Re_*, both monomers show strong density for the FAD cofactor, which is tightly stabilized in its binding pocket through several polar interactions. The conserved and redox-active Cys pair responsible for substrate reduction is located on the *si* face of the isoalloxazine ring of FAD, with the cysteines (Cys39 and Cys44) forming a disulfide bond. The conserved His–Glu ion pair (His442 and Glu447) essential for proton transfer is located at the C-terminal end, positioning it in close proximity to the FAD cofactor of the opposite monomer (Fig. 1 ▸). No electron density was observed for NADPH in the Mtr structures reported in this study, as is often the case for oxidoreductase structures where no NADPH has been included in the crystallization setup. However, the putative NAD(P)H-binding site in Mtr is lined by the conserved amino-acid sequence motif [G*X*G*XX*A/G for the pyrophosphate group of NAD(P)H] commonly found in oxidoreductases utilizing this cofactor (Dym & Eisenberg, 2001 ▸; Hammerstad & Hersleth, 2021 ▸). Part of the HRR*XXX*R binding motif for the 2′ phosphate group of NADPH, found, for example, in *E. coli* low *M*_r_ TrxR (Laurent *et al.*, 1964 ▸), is lacking in Mtr. However, amino-acid substitutions in this motif are commonly seen in NADPH-consuming reductases, and the basic amino acids present in Mtr*_Re_* (for example Arg199 and Arg205) are likely to serve a homologous role in the stabilization of the 2′ phosphate group of the pyridine nucleotide. Moreover, Mtr has previously been shown to be selective for NADPH over NADH, as well as indicating a strict preference for the 2′-phosphate regioisomer when assayed with 3′-NADPH (Patel & Blanchard, 1999 ▸).

### 3.2. Sequence and structure comparison of Mtr with homologous oxidoreductases

The sequence and structure of Mtr were compared with those of other enzymes using different approaches. Structural comparison of Mtr (Mtr*_Re_*) with deposited PDB structures using the *DALI* protein structure comparison server shows that Mtr is highly similar to other disulfide oxidoreductases. Hits with the highest *Z*-score for each of the eight most similar protein types are shown in Table 2 ▸, which correlates to the results from the SSN analysis. High structural similarity is observed to MerA (PDB entry 5x1y; Bafana *et al.*, 2017 ▸), DLD (PDB entry 1ebd; Mande *et al.*, 1996 ▸), GR (PDB entry 6b4o; Center for Structural Genomics of Infectious Diseases, unpublished work), TryR (PDB entry 2tpr; Kuriyan, Kong *et al.*, 1991 ▸), GAR (PDB entry 2rab; Van Petegem *et al.*, 2007 ▸) and TrxR (PDB entry 3dgz; B. E. Eckenroth, R. J. Hondal & S. J. Everse, unpublished work) (r.m.s.d.s of 1.8–2.2 Å), with somewhat lower similarity to quinone reductase (LpdA; PDB entry 1xdi; Argyrou *et al.*, 2004 ▸) and CoADR (PDB entry 5l1n; Sea *et al.*, 2018 ▸) (r.m.s.d.s of 2.7–2.8 Å).

**Figure 3:**
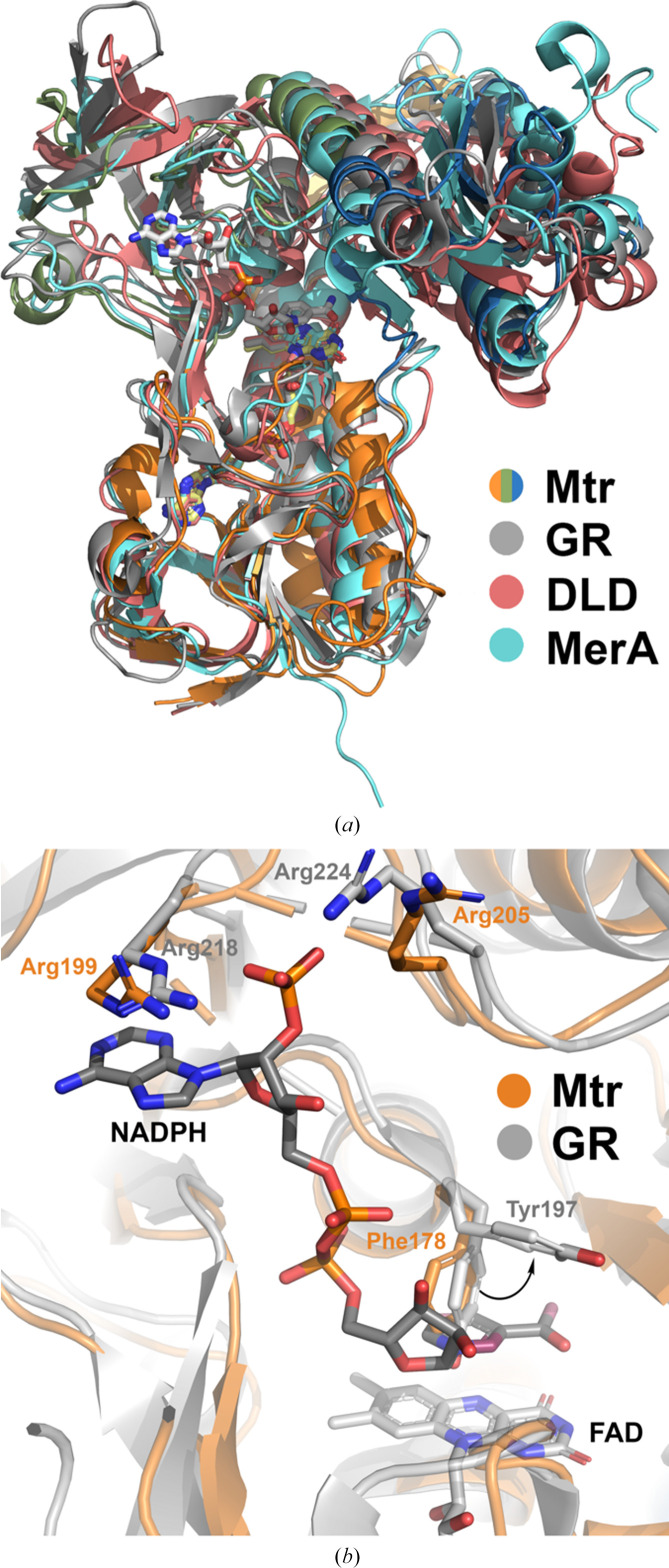
(*a*) Structural alignment of Mtr*_Re_* and the homologous oxidoreductases GR (PDB entries 1gra and 1grb; Karplus & Schulz, 1989 ▸; NADPH from the latter), DLD (PDB entry 2eq9; T. Nakai & N. Kamiya, unpublished work) and MerA (PDB entries 1zk7 and 4k7z; Ledwidge *et al.*, 2005 ▸; A. Dong, M. Falkowski, M. Malone, S. M. Miller & E. F. Pai, unpublished work; NADPH from the latter). Cofactors and the redox-active Cys pairs are represented as sticks and colored by atom type. (*b*) Overlay of Mtr*_Re_* and GR (PDB entries 1gra and 1grb) showing the arginines stabilizing the 2′ phosphate group of NADPH, and Phe178 in Mtr corresponding to Tyr197 in GR which undergoes conformational change when NADPH binds.

The overall fold of Mtr highly resembles that of previously characterized oxidoreductases, as seen from the structural alignment of Mtr with selected homologous structures (Fig. 3 ▸ *a*). All structures share the overall conformation and arrangement of their three domains and the same orientation of the FAD in the FAD-binding domain, as well as the location of the NAD(P)H-binding site. The probable and conserved positioning of NADPH in Mtr can be demonstrated using the coordinates of GR (PDB entry 1grb; Karplus & Schulz, 1989 ▸; Fig. 3 ▸ *b*). A conformational change of an aromatic residue, Phe178, would be required for NADPH binding in Mtr, a rearrangement that has previously been described for GR. In GR, the equivalent Tyr197 on the *re* face of FAD shields the flavin cofactor, blocking the nicotinamide-binding pocket, but rotates away from the isoalloxazine ring of FAD upon NADPH binding (Fig. 3 ▸ *b*), further allowing hydride transfer from NADPH to FAD and ultimately transferring an electron pair to the proximal cysteine of the redox-active Cys pair (Karplus & Schulz, 1989 ▸; Berry *et al.*, 1989 ▸). Hence, by direct comparison, the NADPH nicotinamide ring of Mtr*_Re_* would be stabilized between the *re* face of the isoalloxazine ring of FAD and the phenyl group of Phe178 through stacking interactions. In Mtr, Arg199 and Arg205, which are also conserved in GR (Arg218 and Arg224), are likely to be involved in electrostatic interactions with the 2′ phosphate group of NADPH, which can also be noted as an R*XXXXX*R motif (Figs. 3 ▸ *b* and 4 ▸). The Mtr crystal structures presented in this work confirm that Mtr is composed of the low *M*_r_ TrxR-like fold with an additional C-terminal interface domain, as described for related oxidoreductases such as GR (Hammerstad & Hersleth, 2021 ▸).

To more generally characterize Mtr with respect to other homologous enzymes, a sequence-similarity network (SSN) was generated. The SSN investigation showed that the top ten clusters consisted of other NAD(P)H-dependent disulfide/thiol oxidoreductases containing the canonical low *M*_r_ TrxR fold with an additional interface domain. The clusters were divided into seven groups according to their annotated function (Fig. 5 ▸). Actinobacterial Mtrs cluster together with a group of archaeal dihydrolipoamide dehydrogenases (DLDs; colored orange); the latter is part of a larger group of three DLD clusters (colored red). The close relationship between Mtrs and archaeal DLDs is also seen from the phylogenetic analysis (Supplementary Fig. S1). GRs cluster into a separate cluster, however, containing a few sequences of the related glutathione amide reductases (GARs) and trypanothione reductases (TryRs). Mercuric reductases (MerAs), eukaryotic high *M*_r_ TrxRs and the most distant group of CoADRs each make up distinct clusters, as seen in the SSN and phylo­genetic analysis. CoADRs differ functionally from the remaining enzymatic clusters in view of their single active-site Cys residue used for catalysis and cluster into an independent clade in the phylogenetic tree. Three clusters containing sequences annotated as DLDs, PNDOs and FAD-dependent oxidoreductases or MerAs, with the latter lacking the metal-binding NmerA domain, make up a final miscellaneous group. The SSN shows that the largest groups of homologous enzymes to Mtr are the GRs, DLDs, MerAs and high *M*_r_ TrxRs; however, the closest group that cluster together with Mtrs are the archeal group annotated as DLDs. This close relationship is interesting and will need further investigation to reveal whether there are some additional functional relationships between these groups.

### 3.3. The putative MSSM binding site

Through docking studies and structural comparison with the crystal structure of human GR (with GSSG bound; PDB entry 1gra; Karplus & Schulz, 1989 ▸), the binding of MSSM to the active site of Mtr was examined. Mtr shows high structural similarity to GR, and a similar reaction mechanism to that of GR has been proposed for Mtr in the reduction of MSSM (Patel & Blanchard, 1999 ▸). Therefore, we expected MSSM to bind to Mtr in a similar way as GSSG binds to GR, placing the substrate disulfide in the vicinity of the redox-active Cys pair on the *si* face of FAD, allowing reduction of MSSM. Moreover, as GR is only functional as a homodimer, with each substrate-binding site being formed by both subunits (Schulz *et al.*, 1978 ▸), this is also expected for Mtr.

Initial structure-based blind-docking calculations were performed between homodimeric Mtr*_Re_* and the reduced product MSH (PDB entry 8f5v; Jayasinghe *et al.*, 2023 ▸), returning two significant solutions with Vina scores of −6.8 and −5.8. The MSH molecules are docked into the cavity corresponding to the GSSG binding cleft in GR, placing the MSH thiol near the catalytic Cys pair in Mtr, stabilized by a significant number of putative polar contacts (data not shown).

**Figure 4:**
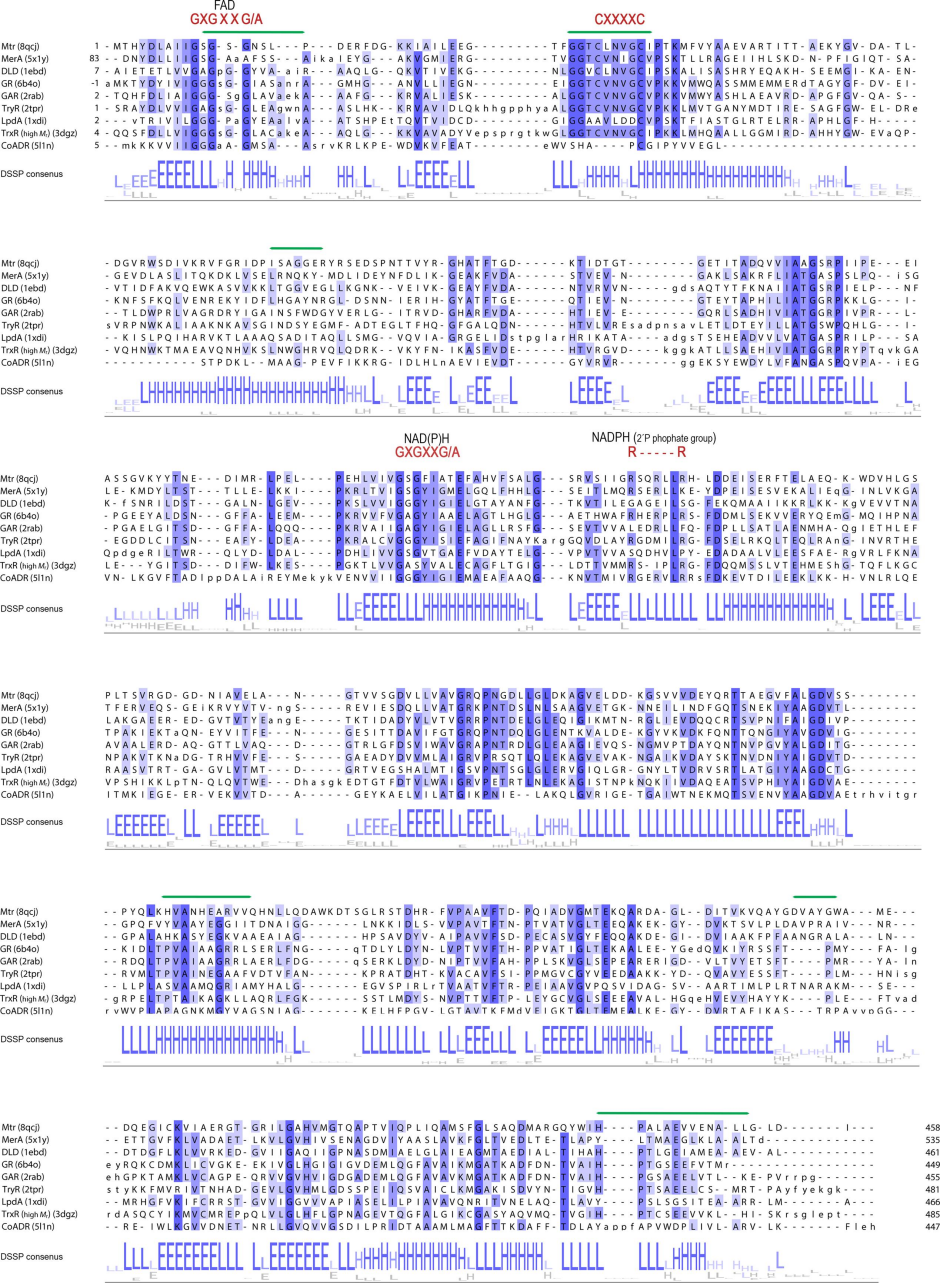
Multiple structural sequence alignment generated by *DALI* for the structures listed in Table 2 ▸. The figure was generated with *JalView* and the sequences are colored according to percentage identity. The consensus secondary-structure assignments by *DSSP* (H/h, helix; E/e, strand; L/l, coil) are shown below the alignment. The characteristic motifs are shown above the sequences and the green lines indicate the residues that line the binding pocket in Mtr and GR.

**Figure 5:**
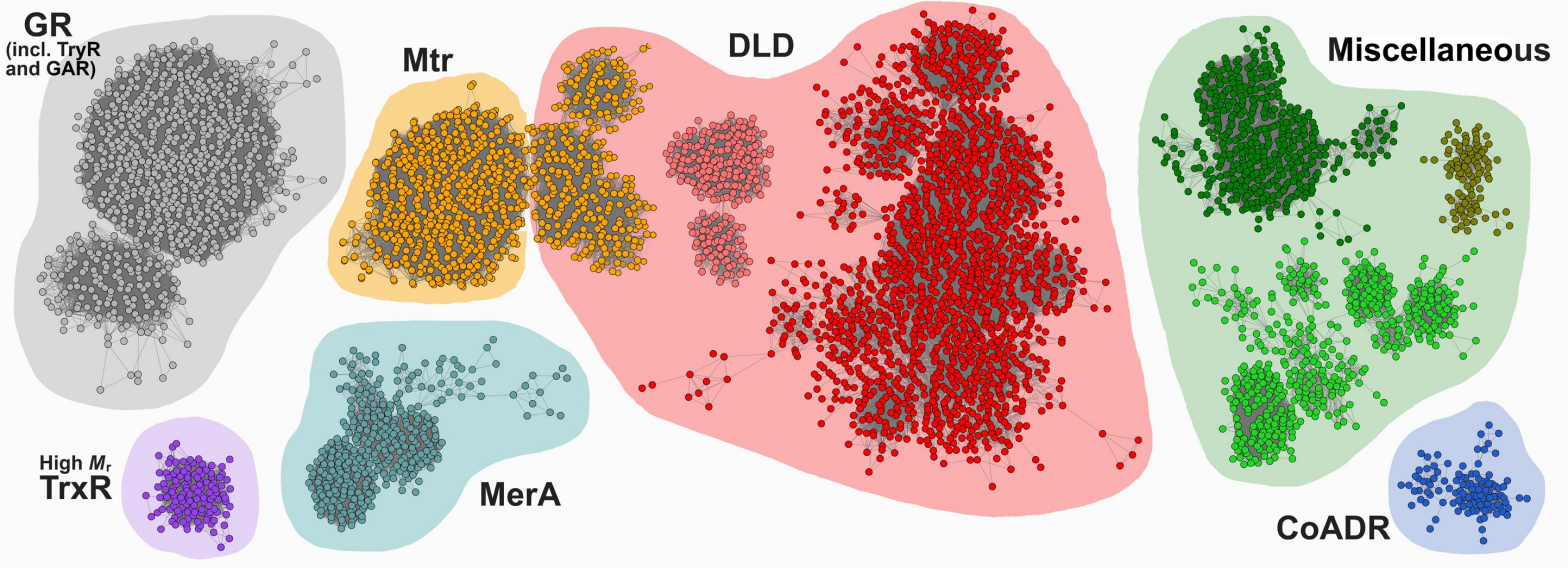
Comparison of Mtr with homologous oxidoreductases through sequence-similarity networks (SSNs). The SSN displays the ten largest clusters. The clusters are individually colored and assembled into seven groups with respect to their annotated function. (GR, glutathione reductases; TryR, trypanothione reductases; GAR, glutathione amide reductases; TrxR, thioredoxin reductases; Mtr, mycothiol disulfide reductases; MerA, mercuric reductases; DLD, dihydrolipoamide dehydrogenases; CoADR, coenzyme A disulfide reductases).

**Figure 6:**
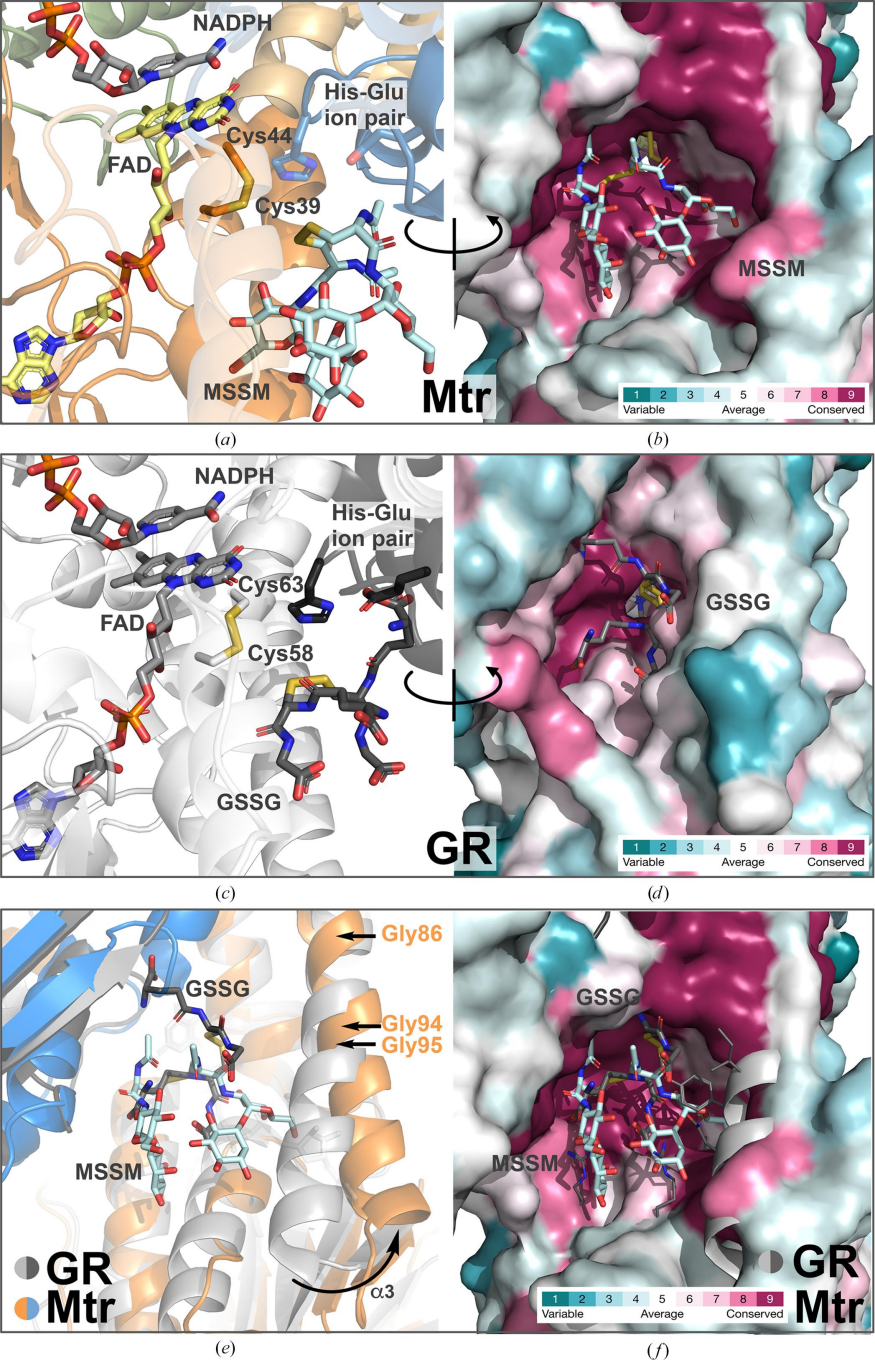
Comparison of the putative MSSM binding site in Mtr*_Re_* (*a*, *b*) with the GSSG binding site from the GR–GSSG complex (PDB entry 1gra; Karplus & Schulz, 1989 ▸) (*c*, *d*). Coordinates for NADPH were taken from PDB entry 1grb (Karplus & Schulz, 1989 ▸). (*a*) and (*c*) show the respective disulfide substrates bound in proximity to the redox-active Cys pairs. The His–Glu ion pairs are located on opposite monomers of the dimers. In (*b*) and (*d*) the active sites are shown from a different angle in surface representation, revealing differences in the space available for substrate binding in Mtr and GR, respectively, and showing the degree of conservation of the residues lining the substrate-binding clefts as evaluated with *ConSurf*. Variable residues are colored turquoise and highly conserved residues are colored maroon. Cofactors, catalytic residues and substrates are represented as sticks and colored by atom type. In (*e*) and (*f*) the binding sites of Mtr and GR are overlaid, showing the increase in the size of the binding pocket that is largely due to a shift of the α3 helix in Mtr compared with GR.

Further docking studies revealed that MSSM can also fit into the expected substrate-binding site with its disulfide positioned in close proximity to the catalytic cysteines of Mtr (Vina score of −7.0; Fig. 6 ▸, Supplementary Fig. S2 and Fig. 4 ▸). This positioning of the substrate would facilitate the initial nucleophilic attack performed by the N-terminal cysteine of Mtr (Cys39) on the MSSM disulfide bond, forming a mixed disulfide, commonly described as the first catalytic step performed by GR and similar FDRs (Fagan & Palfey, 2010 ▸; Deponte, 2013 ▸; Berry *et al.*, 1989 ▸; Pai & Schulz, 1983 ▸; Kallis & Holmgren, 1980 ▸). Analogous to GR, deprotonation of the Cys39 thiol group in Mtr, leading to the formation of this intermolecular disulfide bond, could be accelerated owing to the histidine residue (His442) of the conserved His–Glu ion pair located in the opposite subunit of the Mtr homodimer, which is positioned close to the redox-active Cys pair and FAD cofactor (Figs. 1 ▸ *b* and 6 ▸ *a*). In GR, the interaction between the analogous histidine and glutamate residues has been proposed to facilitate deprotonation in a similar way as in serine proteases, and the histidine has furthermore been suggested to protonate the thiolate leaving group of the first GSH molecule leaving the active site (Pai & Schulz, 1983 ▸; Veine *et al.*, 1998 ▸; Wong & Blanchard, 1989 ▸; Wong *et al.*, 1988 ▸; Arscott *et al.*, 2000 ▸; Fig. 6 ▸ *c*). A nearby tyrosine residue (Tyr114, human GR numbering) was proposed to be involved in assisting the acid catalyst histidine (Krauth-Siegel *et al.*, 1998 ▸); however, this has subsequently been disproved by others (Deonarain *et al.*, 1989 ▸). This tyrosine is, however, only conserved in 9% of GRs in the *ConSurf* search and is not conserved in Mtrs, where it is replaced by a glycine residue (Gly95) that is unlikely to play a catalytic role in the oxidative half-reaction of MSSM reduction.

The α-helix (numbered α3) enclosing one side of the substrate-binding pocket (Trp77–Asp102 in Mtr*_Re_*) is slightly shifted in the Mtr structure (Figs. 6 ▸ *e* and 6 ▸ *f*) compared with GR (PDB entry 1gra; Karplus & Schulz, 1989 ▸), creating a larger binding cavity lined with highly conserved residues (Figs. 6 ▸ *b* and 6 ▸ *d* and Supplementary Fig. S2). Consequently, this allows more space for the larger MSSM substrate to bind, in agreement with the larger size of MSSM compared with GSSG. It is notable that the residues in the α3 helix are more conserved among Mtrs than among GRs (Figs. 6 ▸ *b* and 6 ▸ *d*). That the α3 helix is more straight in GR, while it is bent in Mtrs, could be due to the three glycine residues (known to disorder helices) found in both Mtr*_Re_* and Mtr*_Ms_* but not in GR (Figs. 6 ▸ *e* and 4 ▸).

Overall, our docking calculations provide insight into a likely MSSM binding site in Mtr that is compatible with the expected mechanism for MSSM reduction, supported by functional studies as well as by structural similarity to GR (Patel & Blanchard, 1999 ▸; Holsclaw *et al.*, 2011 ▸; Kumar *et al.*, 2017 ▸; Karplus & Schulz, 1989 ▸).

## 4. Conclusions

The first crystallographic structures of the FAD-containing NADPH-dependent oxidoreductase Mtr, presented in this work, display a homodimeric architecture, assigning Mtrs to the group of oxidoreductases consisting of two dinucleotide-binding Rossmann-like fold domains fused into a single chain: the tDBDFs. As also found in the structurally related GRs, DLDs and high *M*_r_ TrxRs, Mtr contains an additional dimerization domain that is not present in, for example, low *M*_r_ TrxR or Bdr. In contrast to the previously reported asymmetric SAXS structure of Mtr, the crystallographic Mtr structures from *M. smegmatis* and *R. erythropolis* presented in this work display a symmetrical topology, as described for most homologous oxidoreductase structures.

Mtr shares high structural and sequence similarity with GR, the functionally related reductase of GSSG. Although similar overall to GR, the crystal structure of Mtr reveals a larger substrate-binding cleft that is adapted to accommodate the larger and bulkier MSSM substrate. The enlarged, altered and conserved substrate-binding site, partly due to the shifting of a helix, in Mtrs facilitates our proposed binding mode of MSSM, as demonstrated through docking calculations. The shape and size of the binding site may partly explain the previously reported minimum requirement for the glucosamine moiety of MSSM, as well as the lack of GSSG activity (Patel & Blanchard, 1998 ▸, 1999 ▸). The high degree of amino-acid conservation and the binding-site architecture may also contribute to a highly tailored substrate stabilization, possibly contributing to the faster rate reported for the oxidative half-reaction in Mtr (Patel & Blanchard, 2001 ▸).

A large number of tDBDFs comprise oxidoreductases that act on sulfur-containing substrates, and the majority of FDRs belong to this subgroup. FDRs represent a family of enzymes with high sequence and structural homology that catalyze the NAD(P)H-dependent reduction of sulfide-bonded substrates, such as the reduction of thioredoxin catalyzed by TrxRs. The sulfide-bonded substrates also comprise LMW thiols such as GSSG, BSSB, coenzyme A disulfide (CoASSCoA) and MSSM, which are reduced by GR, Bdr, CoADR and Mtr, respectively. The structures of GR, Bdr and CoADR have been described previously, including comprehensive and extensive structural and functional studies of GR and the GSH redox system. No crystallographic data for Mtr have been available to date, creating a knowledge gap in the field of bacterial LMW thiol redox biology. The Mtr structures presented in this work extend our knowledge of Mtrs and tDBDFs, adding an important missing piece to the structural understanding of oxidoreductases and the substrate specificity among reductases of structurally distinct sulfur-containing substrates, in particular LMW thiols. Our structural data, as well as the insight into the MSSM binding mode in Mtrs, may also contribute to future antituberculosis drug development or to new advancements in bioremediation processes, two important areas of research related to actinobacterial survival mechanisms.

## Supplementary Material

PDB reference: mycothiol disulfide reductase from *Rhodococcus erythropolis*, 8qcj

PDB reference: from *Mycobacterium smegmatis*, 8qck

Supplementary Figures. DOI: 10.1107/S205979832400113X/gi5042sup1.pdf

Diffraction data.: https://doi.org/10.15151/ESRF-DC-1468846919

## Figures and Tables

**Table 1:** Crystallographic data-collection and refinement statistics Values in parentheses are for the outer shell.

	Mtr*_Re_*	Mtr*_Ms_*
Data collection		
X-ray source	ID23-2, ESRF	ID30B, ESRF
Detector	PILATUS3 2M	EIGER2 X 9M
Temperature (K)	100	100
Wavelength (Å)	0.87311	0.91840
Space group	*P*3_1_	*C*222_1_
*a*, *b*, *c* (Å)	92.9, 92.9, 100.2	51.9, 209.6, 241.9
α, β, γ (°)	90, 90, 120	90, 90, 90
Rotation	Standard	Standard
Rotation range per image (°)	0.1	0.2
Total rotation range (°)	360	180
Exposure time per image (s)	0.025	0.02
Flux (photons s^−1^)	8.8 × 10^10^	1.1 × 10^12^
Transmission (%)	6.2	10
Beam size (µm)	4 × 4	10 × 10
Crystal size (µm)	100 × 50 × 50	200 × 30 × 30
Average diffraction-weighted dose (MGy)	9.7	27.0
Mosaicity (°)	0.09	0.17
Resolution range (Å)	46.5–2.90 (3.08–2.90)	49.4–4.70 (5.25–4.70)
Total No. of reflections	219051	44357
No. of unique reflections	21492	7284
*R*_merge_ (%)	20.9 (172.4)	63.5 (118.3)
*R*_p.i.m._ (%)	6.9 (56.1)	27.8 (51.4)
Completeness (%)	100 (100)	99.9 (99.9)
Multiplicity	10.2 (10.3)	6.1 (6.3)
〈*I*/σ(*I*)〉	10.1 (1.7)	3.0 (1.6)
CC_1/2_	0.997 (0.593)	0.942 (0.847)
Refinement		
*R*_work_/*R*_free_ (%)	17.7/22.3	29.3/33.8
Mean overall *B* factor (Å^2^)	79.0	113.0
Wilson *B* factor (Å^2^)	69.0	25.8
Asymmetric unit content	Homodimer	Homodimer
Protein residues in sequence	458	461
Total modeled residues in asymmetric unit		
Protein residues by chain	*A*, 458; *B*, 458	*A*, 461; *B*, 461
Ligands by chain	*A*, 1 FAD; *B*, 1 FAD	—
Matthews coefficient (Å^3^ Da^−1^)	2.5	3.3
Solvent content (%)	51.1	62.9
Ramachandran plot		
Favored (%)	95.7	81.4
Allowed (%)	4.9	14.4
Outliers (%)	—	4.2
R.m.s.d., bond lengths (Å)	0.003	0.003
R.m.s.d., bond angles (°)	0.752	0.826
Estimated coordinate error (Å)		
Based on Luzzati plot	0.51	1.24
Based on difference between *F*_obs_ and *F*_calc_	0.55	1.23
Based on diffraction-data precision index	0.38	1.57
PDB code	8qcj	8qck

**Table 2:** Structural comparison of Mtr*_Re_* with the eight most similar enzyme classes from the *DALI* search

Protein	*Z*-score	R.m.s.d. (Å)	Residues	Aligned residues	Sequence identity (%)	PDB code
MerA	45.5	1.9	452	441	30	5x1y
DLD	44.5	2.0	455	444	27	1ebd
GR	43.3	1.9	450	434	29	6b4o
GAR	42.9	2.2	451	436	31	2rab
TryR	41.1	2.0	481	440	26	2tpr
LpdA	40.7	2.8	459	436	24	1xdi
TrxR (high *M*_r_)	40.5	2.1	482	437	26	3dgz
CoADR	33.8	2.7	444	401	21	5l1n
