# Supplementary material for: The crystal structure of mycothiol disulfide reductase (Mtr) provides mechanistic insight into the specific low-molecular-weight thiol reductase activity of Actinobacteria
Source: Acta Crystallogr D Struct Biol. 2024 Feb 19;80(Pt 3):181–93. doi: 10.1107/S205979832400113X (PMC10910545; doi:10.1107/S205979832400113X)
Supplement: Supplementary file 1 [file d-80-00181-sup1.pdf]

Acta Cryst D
STRUCTURAL BIOLOGY

Volume 80 (2024)

Supporting information for article:

The crystal structure of mycothiol disulfide reductase (Mtr) provides mechanistic insight into the specific low-molecular-weight thiol reductase activity of Actinobacteria

Javier Gutiérrez-Fernández, Hans-Petter Hersleth and Marta Hammerstad

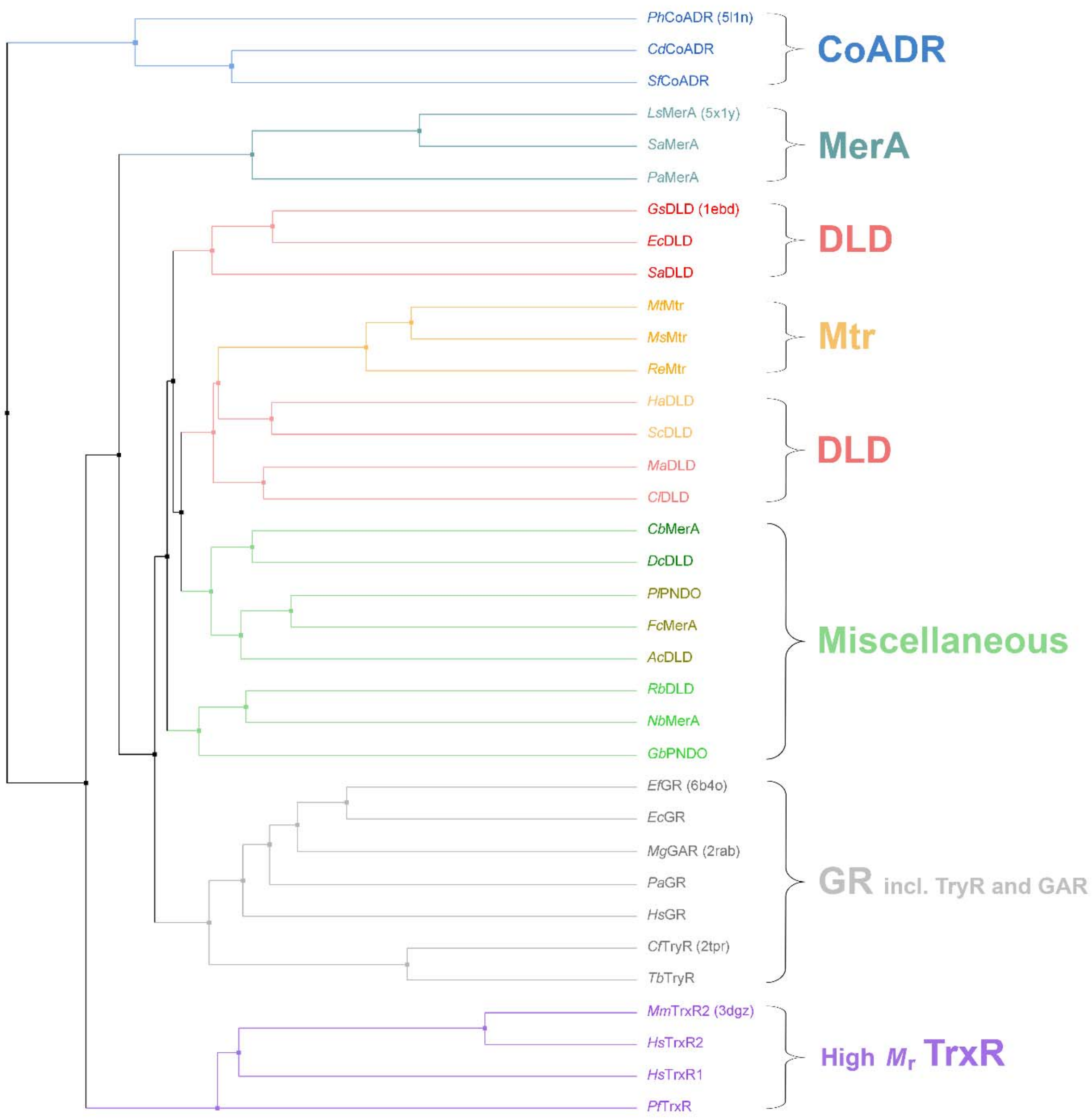

Figure S1 Phylogenetic tree analysis of selected oxidoreductases from the different SSN clusters in Figure 5 and Table 2. Coloring according to Figure 5. The PDB entries are listed in parenthesis for the oxidoreductases from Table 2. The organisms used were: *Ph*: *Pyrococcus horikoshii*; *Cd*: *Clostridioides difficile*; *Sf*: *Streptococcus ferus*; *Ls*: *Lysinibacillus sphaericus*; *Sa*: *Staphylococcus aureus*; *Pa*: *Pseudomonas aeruginosa*; *Gs*: *Geobacillus stearothermophilus*; *Ec*: *Escherichia coli*; *Mt*: *Mycobacterium tuberculosis*; *Ms*: *Mycobacterium smegmatis*; *Re*: *Rhodococcus erythropolis*; *Ha*: *Halobacteriales archaeon*; *Sc*: *Sulfidibacter corallicola*; *Ma*: *Methanotrichaceae archaeon*; *Cl*: *Candidatus lokiarchaeota*; *Cb*: *Chloroflexota bacterium*; *Dc*: *Deinococcus cavernae*; *Pl*: *Peribacillus loiseleuriae*; *Fc*: *Faecalicatena contorta*; *Ac*: *Amycolatopsis camponoti*; *Rb*: *Rhodospirillaceae bacterium*; *Nb*: *Nitrospinota bacterium*; *Gb*: *Gammaproteobacteria bacterium*; *Ef*: *Enterococcus faecalis*; *Mg*: *Marichromatium gracile*; *Hs*: *Homo sapiens*; *Cf*: *Crithidia fasciculata*; *Tb*: *Trypanosoma brucei*; *Mm*: *Mus musculus*; *Pf*: *Plasmodium falciparum*.

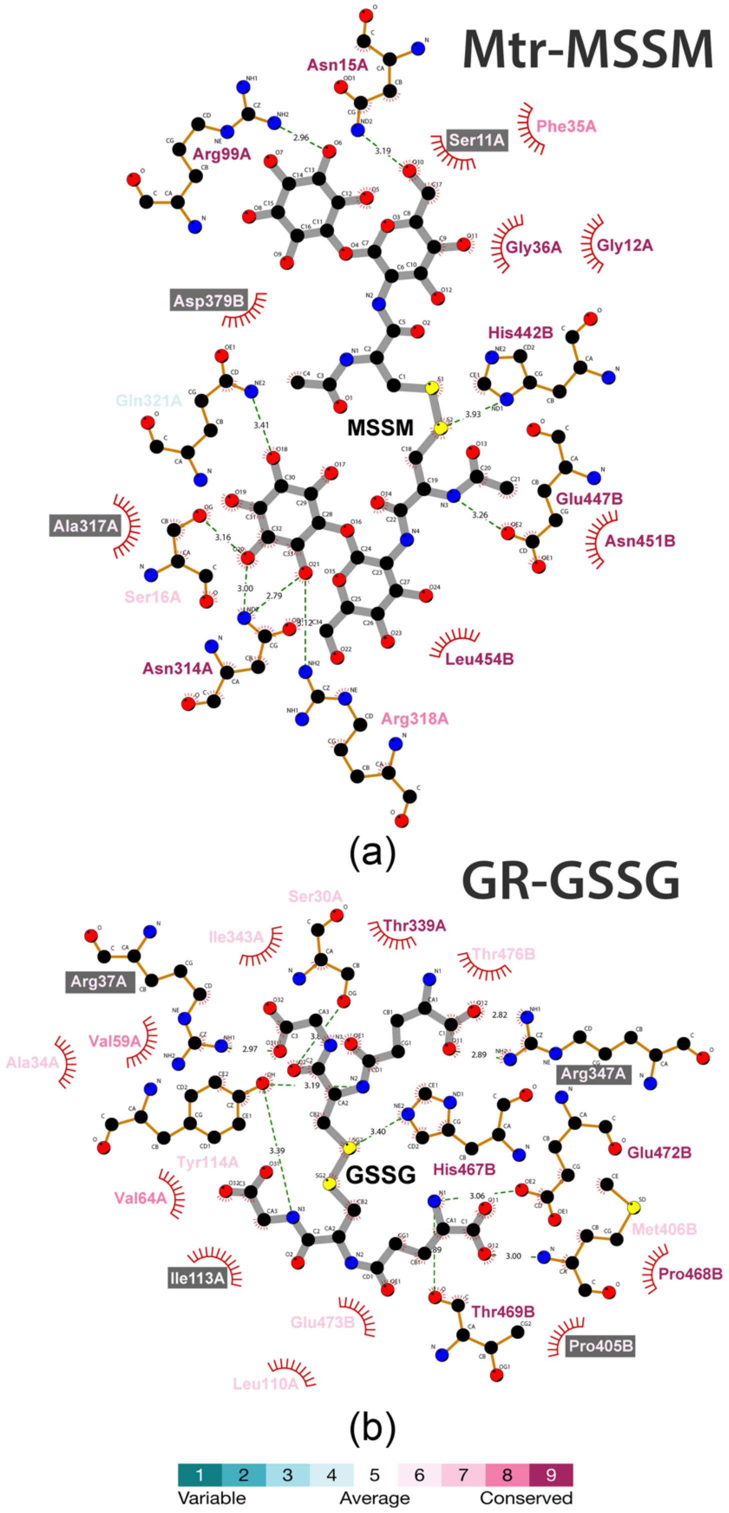

Figure S2 LigPlots showing the binding sites of LMW thiols in Mtr and GR, with the respective residues interacting with the ligands. (a) Potential binding site of MSSM in Mtr_{Re}, obtained from molecular docking calculations. (b) GSSG binding in human GR (PDB entry 1gra (Karplus & Schulz, 1989)). Residues lining the binding sites are evaluated using ConSurf, and colored accordingly, with variable residues colored in turquoise and highly conserved residues colored in maroon.
